# The approaches Hong Kong Chinese mothers adopt to teach their preschool children to prevent influenza: a multiple case study at household level

**DOI:** 10.1186/s12912-016-0172-4

**Published:** 2016-09-01

**Authors:** Winsome Lam, Cathrine Fowler, Angela Dawson

**Affiliations:** 1 School of Nursing, The Hong Kong Polytechnic University, Hong Kong, China; 2 Faculty of Health, University of Technology Sydney, Sydney, Australia

**Keywords:** Parental teaching, Seasonal influenza, Parent-child interaction

## Abstract

**Background:**

In Hong Kong, the population is at risk of seasonal influenza infection twice a year. Seasonal influenza is significantly associated with the increased hospitalization of children. Maintaining personal hygiene and vaccination are the most effective measures to prevent influenza infection. Research demonstrates a positive relationship between the health practices applied by parents and the behaviour of their children highlighting the importance of parental heath education. However, there is minimal research that provides an understanding of how Hong Kong Chinese parents teach their children to prevent seasonal influenza.

**Methods:**

Mixed methods research was undertaken that employed a multiple-case study approach to gain an understanding of parental teaching practices regarding seasonal influenza prevention. Purposive intensity sampling was adopted to recruit twenty parents and their healthy children. A thematic analysis was employed to examine the qualitative interview data and the quantitative survey data were examined descriptively. These data were then integrated to provide a more rigorous understanding of parental teaching strategies. Comparisons were made across cases to reveal commonalities and differences.

**Results:**

Five major themes were identified: processes parents used to teach personal hygiene; parent-child interaction during teaching; approaches to managing children’s health behaviours; enhancing children’s healthy practices; and parents’ perspective of the role of the nurse in health promotion.

**Conclusions:**

This study provided valuable insight into the approach of Hong Kong Chinese parents in teaching their children to prevent seasonal influenza. The results indicate that parents can be better supported to develop effective strategies to teach their preschool children hygiene practices for seasonal influenza prevention. Partnerships with community nurses can play a role in building effective parent-child interactions to enhance children’s learning and adoption of healthy practices.

## Background

In Hong Kong, influenza is usually common in the periods from January to March and July to August [1]. The virus is transmitted from person-to-person through droplet and direct contact [1, 2]. Yearly influenza epidemics can seriously affect all age groups but particularly those with immature or compromised immune systems such as young children and the elderly with chronic illness [1, 3]. The viruses causing influenza are constantly changing. Exposure to these new virus variants can potentially lead to influenza epidemics [2, 3]. Maintaining personal hygiene including proper hand washing, cough etiquette and regular seasonal influenza vaccination uptake [1, 4] are recommended as the most effective measures to prevent the spread of influenza [3, 5].

Research demonstrates a positive relationship between the health practices used by parents and children’s behaviour [6]. A study by Song et al. (2013) demonstrates that parents’ hand washing practices are significantly correlated with children’s hand hygiene practice and research suggests that the hand hygiene behaviour of adults is likely to be the result of lessons learned in childhood [7]. Young children spend extended periods of time with their parents ideally positioning parents, through their daily interactions, to teach and reinforce their children’s health knowledge and practices. Without family involvement, health practices are unlikely to be sustained once the health promotion intervention ceases [8].

The preschool period is recommended as the most suitable time for parents to initiate and support their children to develop lifelong health behaviours [9]. Preschool children learn health knowledge and practices as a result of parental modelling and repeated opportunities for practicing these health behaviours [10, 11]. Parents assist children internalize these health practices into their daily behaviours through repeated interactions embedding the behaviours as lifelong habits. These regular practices have also demonstrated lasting effects on subsequent adult activity-related behavioural patterns [12].

The cognitive and functional abilities of preschool children are still developing [10, 11]. Their ability to manage and remember a large amount of health knowledge and practices at a single interaction may be challenged. To develop competence children often require information to be repeated and several opportunities to practice tasks along with appropriate encouragement [13]. The effectiveness of parental teaching depends on the extent to which this teaching has advanced a child’s understanding of the health practice [14]. A child’s level of understanding will impact upon their health practice compliance. If parents can enhance children’s understanding of health practices such as correct hand washing compliant, the risk of preschool children contracting infectious diseases such as influenza will be lowered. Using a teaching process that includes: alerting the child to a teaching moment, providing visual cues and verbal instruction, giving encouragement and providing feedback will assist both the child and parent [15]. Importantly, parental teaching strategies assist in the effectiveness of children’s learning and the enhancement of the parent-child interaction through one or two way communication [16]. In addition, providing clear and consistent messages to the child, allowing time to question, and appreciating the child’s efforts through the provision of feedback have also been found to be useful [10, 11].

A review of the literature identified that most research studies are focused on the association of parenting styles either related to healthy lifestyles such as healthy eating [17, 18] or health practices such as internet use [19]. There is minimal research investigating the teaching approaches of parents, particularly Hong Kong Chinese parents regarding how they teach their preschoolers to prevent seasonal influenza. It is therefore worthwhile investigating how parents and their preschoolers interact to develop health practices and build prevention knowledge. The aim of this study is to provide an insight into parents’ roles and teaching approaches when supporting their children’s development of health practices, particularly those to prevent influenza occurring within the household.

## Methods

This mixed methods research study employed a multiple-case approach [20, 21] to gain an understanding of parents’ health teaching practices and their interaction with preschool children regarding seasonal influenza prevention. A case comprised a parent with a preschool child between 3-to-5 years. The approach allowed the researchers to analyze phenomena from multiple perspectives within each real-life setting and across settings to understand the similarities and differences between the cases; discover previously unknown features regarding culture specifically Chinese parental teaching and parent-child interactions related to seasonal influenza prevention [22, 23].

### Recruitment and participants

Purposive intensity sampling was used to recruit 20 Hong Kong Chinese parents and their healthy preschool children aged from 3-to-5 years old. A networking approach was used to identify potential preschools. Letters and study information sheets were sent via the person-in-charge of five Hong Kong kindergartens for distribution to Chinese parents, inviting them to participate in the study by contacting the researcher. Study participants were ultimately enrolled from three kindergarten schools. The researcher then personally invited the parents and provided a detailed explanation of the research. The selection criteria were parents with healthy 3-to-5 year old children who were willing to communicate and share their experiences. In total, 23 parents were contacted and 20 parents agreed to participate in this study.

Eleven families were living in private apartments and nine families were living in public estates. Nine families had three generations: grandparent, parent and child living in the same household. Eleven families had two generations: parent and child living in the same household.

### Data collection

Data collection was conducted by a researcher with a history of clinical experience in family health education, health promotion, and skills in conducting family interviews. Data were obtained from multiple data sources. These sources included: a semi-structure interview guide, an observational checklist and a demographic survey. The in-depth semi-structured interviews were carried out first. Open-ended questions were used to elicit participants’ teaching strategies and included “what concerns, issues or challenges do you face when trying to encourage your child to prevent influenza and carry out healthy practices” and “what are the strategies/activities you use at home to promote your child’s healthy behaviours or minimize the risk of developing influenza”. The responses were digitally recorded and transcribed verbatim. As part of the interview phase, closed questions in a survey format were used to collect participants’ demographic data.

The mothers were then invited to demonstrate the procedure of teaching their children to wash their hands and wear a facemask. The mothers were not provided with any instructions or prompting on how to undertake this task by the researcher during the observation. The aim of this data collection technique was to identify the mother’s existing knowledge and skills in teaching and supporting her child’s hand washing technique. Maternal teaching strategies and parent-child interactions during the demonstration were observed and recorded using an observational checklist. A framework was used to guide the observations that involved identifying the steps parents took when teaching hand washing and facemask wearing [15]. These steps were: alerting the child to the activity to be learned or practiced; instructing the child to perform what is expected or the steps to be taken; allowing the child to demonstrate; evaluating the standard of the child’s health practice; and providing feedback to the child on their health practice performance [15]. The observations of parent-child interaction involved: one or two way communication; clear and age appropriate task instructions; showing appreciation of the task attempts; providing encouragement when encountering something difficult; and language style used by the parent [15]. Both teaching strategies and the characteristics of parent-child interaction were assessed using a dichotomous scale (yes/no) with a space for reflective notes to be written on each item.

The demographic survey, semi-structured interview guide and observational checklist were validated, using a content validity index with four-point scale ranging from 1-to-4 (not relevant = 1, some relevance = 2, very relevant = 3, extremely relevant = 4), by a panel of academic experts, community nurses and school teachers who were knowledgeable about infection control and child and family health. This index ensured that the questions were technically correct, unambiguous, culturally appropriate and able to provide adequate information to address the proposed research questions [24].

### Data analysis

The process of data analysis was conducted concurrently with data collection, as constructs from the previous interview guided the collection of data in the next interview. The tape was replayed within 1 or 2 days after the interview with the researcher carefully listening to the questions and the tone of the responses. Each taped interview was transcribed verbatim from Cantonese to English. All communication forms including laughter, pauses in the conversation and expletives were also included in the script. After the transcription, the whole script was checked against the tape for accuracy. Back translation of the quotes from English to Cantonese, were completed to ensure the validity of qualitative data. The concordance between the interview and observation data enhanced the rigor of the findings [21].

A thematic analysis of the interview transcripts was employed to categorize, tabulate and examine the data to address the propositions of the study [22]. First, the raw data from the 20 semi-structured interviews were reviewed to mark and link primary codes according to the research objectives. These codes were grouped into broad categories through repetitive scanning of the data [22]. The categories were then collated into theme clusters. Analysis continued until no new themes emerged [21, 25, 26]. Data saturation was observed when similar meanings and categories were noted from the data after the eighteenth interview. Two additional interviews were completed to confirm that the mothers were not providing new data.

A deductive approach was then, applied for pattern-matching [21]. The researcher went back to the original data, read through it repeatedly and compared the derived patterns with previously outlined predefined patterns in order to ensure no missing data and consistency with the study objectives. The themes and data extraction were independently examined by two other researchers in order to achieve an agreement on pattern matching for the purpose of internal reliability of the study [22]. Exclusions to the thematic codes were also discussed with these researchers.

The demographic and numerical data from the surveys and observational checklist revealed an overall understanding of parental teaching strategies and the interaction with their children in seasonal influenza prevention and related practices. These quantitative data enriched the qualitative analysis of each case as it provided a context for parents’ responses.

In the final stage, a comprehensive comparison was made across cases including families’ demographic information, living environment, parental education level, parental teaching strategies and the characteristics of parent-child interaction in relation to hygiene practices. This cross-case analysis of the multiple cases provided an understanding of the commonalities and differences between cases regarding hygiene practices for seasonal influenza prevention [22, 26].

## Results

Twenty Hong Kong Chinese mothers agreed to be interviewed. Nineteen mothers and their preschool children participated in the hand washing and facemask wearing observation component of this research. One of the mothers did not participate in the observation due to reluctance to be involved in the demonstration. All mothers were the main caregivers of their children, aged between 28 and 45 years. Ten mothers had completed secondary school education and ten completed tertiary education. Fifteen mothers were housewives and five mothers were full-time workers with their children being cared for by other family members (Table 1).

**Table 1:** Personal particulars of 20 participants

Characteristics	Number
Parent gender	
- Female - Male	20 0
Parent age	
- 28 years old - 30-to-35 years old - 36-to-40 years old - 41-to-45 years old	2 7 8 3
Education level completed	
- Secondary school level - Tertiary level	10 10
Employment status	
- Housewife - Full time workers	15 5
Child’s main caregiver	
- Mothers - Both father and working mother - Grandmothers and working mother (shared)	15 1 4

The qualitative analysis of the interviews identified five major themes within the data. The themes were: processes used by parents to teach personal hygiene; parent-child interaction during teaching; approaches to managing children’s health behaviours; enhancing children’s healthy practices; and parents’ perspective of the nurse’s health promotion role. The quantitative data gleaned from the survey and observations enriched the interview findings.

### Processes used by parents to teach personal hygiene

The mothers reported that they did not use any specific teaching method to teach their children health practices. They sometimes kept silent during the observation session and did not provide feedback if their child was able to perform the health practices correctly.

If they [child] wear mask properly, the wire at upper side, I seldom provide feedback [on their health practices]. I only keep silent. (P1-M89)

I do not have specific method or steps to teach them [health practices]. (P11- F292)

The findings of the above quotes were consistent with the data observed from handwashing and face masks demonstrations. Most mothers did not fully demonstrate the five teaching steps listed on the observation checklist during the hand washing and facemask wearing sessions. All of the 19 mothers directly asked their children to participate in hand washing and facemask wearing. Seven participants gave instructions with explanations to their children concerning what was expected, or the steps to be taken in hand washing. The other 12 mothers kept silent during the hand washing procedure. Twelve parents allowed their children to demonstrate hand washing and facemask wearing and provided sufficient time for the procedures to be completed without intervening. Seven participants allowed their children to start demonstrating the procedures. However, once their children encountered difficulties, the mothers intervened by helping their child complete the task without asking any probing question or allowing extra time to independently problem-solve. After finishing the hygienic practices, twelve mothers evaluated the child’s standard of hand washing and facemask wearing according to the local government procedure guides. The other seven parents kept silent and did not evaluate either the standard of hand washing or facemask wearing. Only one mother provided feedback such as “you do well by yourself this time and keep on this practices” to her child on the performance of hand washing and facemask wearing (Table 2).

**Table 2:** Parental teaching process of hand washing and facemask wearing

Parental teaching process of 19 participants	Yes	No
Alerts child to the activity	19 (100 %)	0
Instructs child to perform what is expected	7 (33.84 %)	12 (63.16 %)
Allows child to demonstrate	12 (63.16 %)	7 (33.84 %)
Evaluates the standard of child’s health practice	12 (63.16 %)	7 (33.84 %)
Provides feedback to child on their performance	1 (5.26 %)	18 (94.74 %)

### Parent-child interaction during teaching

Two characteristics identified during the qualitative interviews were praising children when the children: performed a task that mothers felt had exceeded their child’s abilities, and if they felt their children required limited parental guidance.

I will only praise her when they do something good…such as if the tasks exceed her abilities, but they can do it well. Then, I will praise them. For example, under this situation, I will scold her. She misread the sign of plus and minus in mathematic calculation. Then I will scold her. (P11-F361)

If there is something he is able to do for example washing his hands before eating, I won’t praise him. It depends on situation. If the thing is far from his ability and I think he cannot do it but turns out he does it well. Then I will praise him. (P18-M374)

Limited parental guidance on children’s health practices was observed in the following quote.

I seldom talk to or teach my son how to wear the mask properly. They elicit most of the things by themselves. I only correct them when they wear the mask wrongly. (P1-M87)

I will only teach her when she cannot do it. If she says, ‘mum, I do not know how to do it, I do not know how to fit it, can you teach me? Then I will teach her. (P8-M377)

The findings of the above quotes were consistent with the characteristics observed during the observation of hand washing and facemask wearing. All of the 19 mothers engaged their children in conversation when commencing the procedure performance. Questioning and answering skills were used to check children’s understanding. When two children interrupted their parents’ conversations, two of the mothers stopped talking and listened to their children’s requests before proceeding to the next step. Twelve mothers used language that appeared to be clear enough for their children to understand their instruction. Mothers described different aspects of hand washing when carrying out teaching to enhance their child’s learning including: the location of areas on the hand to be cleaned, and hand actions used to effectively clean the hands and the colour and shape of the face mask when guiding facemask wearing. Seven mothers observed their child’s practices without any explanation or description of the actions. Five mothers verbally appreciated their child when the procedure was correctly completed. Fourteen mothers remained silent when their child completed the procedure appropriately. Five children encountered difficulties during hand washing and facemask wearing. Two of the five mothers encouraged their child when they had difficulties and three remained silent. All mothers used a combination of commands and explanations when asking their child to do or not to do the health practices.

### Approaches to managing children’s health behaviours

The mothers reported various strategies to ensure their child followed their instructions and maintained healthy behaviours. These included: punishing the child, threatening them and giving incentives. Physical punishment and scolding were two methods regularly identified by the mothers to regulate children’s misbehaviours.

I also think physical punishment is … [of the] same important as praising to children. If she is naughty, I will hit her butt or her hands with cane or bare hands. Sometimes I will punish her by asking her to stand which allows time for her to calm herself. (P15-P457)

I really ask her [child] a lot of times… for example, do not use her hand to touch her feet and I did it with explanation, but she still does not listen. She still does not change. I am out of solution. I did everything. I warned her once, I hit her hands once. (P8- M161)

When he does not listen to my instruction, I will not allow him to do something he wants to do. If he does something related to health practice wrong, I will scold him. If he still doesn’t listen, I will punish him when we go home. (P9-M112)

The mothers claimed using a threatening approach and providing consequences was an effective way to stop their children’s misbehaviours.

When he doesn’t follow my instruction, I sometimes will tell him, “If you do not do it, I will not take you out to play”. It’s effective. In general, when we tell him this, he will be scared and will follow our instructions. (P12-M336)

I let my daughter see the photos of ugly fingers and told her this is the consequences of biting nails. After seeing the photos, she got scared, and then she stopped biting. I have no other ways since she did not listen to me. Therefore, I have to intimidate her. (P14-M399)

Most of the mothers used incentive such as gifts, snacks and playing to encourage or sustain children’s health practice. The following quote provides an example of an incentive that was provided.

If he does it well [correct health practices steps], I will give him a stamp every time as a reward. When he collects a certain amount of stamps, he can use the stamps to exchange a present from me. His health practice has improved. (P19-M76)

### Enhancing children’s health practices

During the interview, the mothers identified singing and storytelling as useful pedagogies to encourage and sustain their child’s health practice.

When he is not willing to wash his hands, I will sing the “hand washing” song with him. Then, he will wash it. If the thing he is willing to, I will do it more often. Just like singing the song can help him to wash his hands more often. (P4-M190)

Taking story-telling as an example, I will ask her some questions in between story telling. I will ask her whether the behaviour showed in the storybook is correct or not, is it good to do so, or will you do that? (P5-M405)

### Parents’ perspective of the role of the nurse in health promotion

Mothers claimed that the community nurse could provide explanations and advice to families and their children on health related issues. The nurse could further expand their role by assisting families apply new knowledge and skills they have developed into disease prevention within the context of their home and local community. Some mothers believed that information provided by nurses would be more convincing. The following quote supports the greater involvement of nurses providing health education as the mothers felt that their children and older relatives would be more likely to follow what a nurse advises them to do.

Community nurses in the community can teach everyone about the prevention of flu. For example, when having flu symptoms and we do not want to take medication, we can ask [community nurse] what we can do to prevent this. At least, nurses have the knowledge compared to normal people. By asking them, they can explain more and give advice to us, this is much better. (P8- M283)

I will use the knowledge obtained from the community nurse [to prevent disease/illness] since they have more knowledge on prevention of influenza and can teach us. Also they [community nurses] are more convincing. If I am the one who talk to elder people [in my family], they will ignore me. But, if there is a professional nurse, they may listen. (P11- F249)

In the first quote, the mother emphasized the importance of health consultation role of nurses and teachers within the community. In the second quote, the mother claims that knowledge transfer related to disease prevention from the nurse to the mother might happen during nurse-parent and nurse-family interaction, in particular, older family members.

In the next two quotes, there is a shift in focus as the mothers provide an explanation of the importance of the nurses’ and teachers’ roles in health education provision.

They [children] may not remember even you say it for ten times to them. But if a community nurse comes to tell them, children will remember. I think it should be effective for community nurses to go to kindergarten or primary school to promote health, particularly for kindergartens. (P17-M265)

I think children always listen to nurses. Just like they listen to their teachers. I think if the talk is held by nurses, it will be more effective. The children will listen to what nurse says. (P19-M317)

In the above quotes, the mothers identified an opportunity for the nurse to provide health promotion during the child’s attendance at kindergarten. In the final quote, the mother justified the nurse’s role in that children always listen to nurses, providing the nurse with a level of authority as a health educator.

## Discussion

The findings from this study demonstrate that none of Hong Kong Chinese mothers in this study applied the whole teaching process as per our framework of five identified steps when teaching their preschool children hygiene practices to promote health and prevent seasonal influenza. Some parents adopted controlling approaches to ensure their children’s compliance in health practices or provided incentives such as gifts or snacks. The strategies parents applied may not be optimal and therefore this study provides insights that could assist nursing service planners to design child and family health promotion programmes to better support parents to guide the development of their children’s healthy behaviours.

### Enhancing effective parent-child interaction and teaching for health

The key determinant for successful implementation of health education programmes is the educator’s, or in this case the parent’s ability to communicate effectively [27]. In recent years, the focus of prevention research has shifted from the parent and child as individuals to the parent-child dyad [16]. This reciprocal interaction relies on the level of a child’s cue clarity, a parent’s responses and a logical interaction sequence to ensure effective communication [15]. Our study provides observational evidence of communication issues where reciprocal interaction could be enhanced. We found that while 12 mothers used language that was clear enough for their child to understand, the hand washing instructions given by seven mothers’ were not understood by their children. The clarity of instruction and quality of information provided to the children could be improved by assisting new mothers to learn the required steps of practice, such as hand washing, in a systematic way.

Positive parent-child communication is identified as contributing to effective health practice learning [27]. It enables parents to support their children’s health behaviour regulation, maintain involvement and act as a positive role model for their children [28]. It reduces the need for the mother to rely on assertive and aversive strategies to increase child health practice compliance [16]. In this study, punitive strategies were found to be commonly employed as a method to gain their child’s compliance. Some mothers identified a lack of confidence to change their children’s behaviour. Therefore enabling parents to build confidence and effective communication with their children is central to health promotion interventions to enhance child health practice learning [27].

Interventions to support the adoption of healthy behaviours involve assisting parents to improve their knowledge and modelling skills. Practice sessions involving both parents and their children in demonstrations of these health practices are a key way forward. Parents can be supported to develop effective communication skills, knowledge about their children’s physical development and ways of learning. Scenario based learning involving cough etiquette or hand washing can guide parents and children during practice sessions. Through these hands-on interactive activities children are provided with opportunities to apply their new knowledge into everyday practice enabling parents to develop confidence [29, 30] in guiding child health behaviour. With the support and consultation of community nurses, inappropriate and ineffective concepts and practices related to child learning and interactions can also be corrected and positive practices consolidated. Nurses can facilitate these practice sessions through face-to-face education and phone consultations or teleconferences [31, 32], these strategies can provide parents with additional feedback and support post intervention.

### Integrating parental teaching skills in child and family health education

In this study, some mothers who adopted a controlling approach to regulate children’s health behaviours claimed they had no other way to manage their child’s behaviour. Chinese parents have been reported to use controlling and restrictive approaches in child rearing [33]. Children may be required to be unquestionably obedient to their parents who may influence the use of such parenting approaches. Frequent use of restrictive or controlling approaches by parents have been associated with children’s behavioural problems such as internet misuse [34] and unhealthy eating [16]. Health professionals can support parents to employ teaching approaches to adopted healthy behaviours that encourage children using strategies that promote and celebrate child achievements [35].

### Nurse-parent-child partnerships in health promotion

Parental expectations concerning nurses’ and teachers’ roles in health education were also highlighted in our study [36]. A multi-faceted approach involving the health sector, community partners and families in health promotion is a fundamental goal of health promotion [37]. Partnerships between community nurses and teachers provide opportunities to strengthen parents’ abilities to create healthy households [38] by appropriately using health services, to assist parents set achievable goals [39] and provide emotional support and information. This partnership is of particular importance to child rearing ensuring that families are actively involved in enhancing their health [40]. It is vital for nurses to foster relationships early on with parents and their child by: involving parents and children in conversations about health promotion [41]; communicating the parents’, child’s and nurse’s expectations with regard to learning; and encouraging parents to model health behaviours and practices for their children [28]. These approaches will assist in maintaining an optimal environment for health promotion and illness/disease prevention at both community and household levels.

## Limitations

Further experimental studies are warranted to examine the impact and effectiveness of parental training on the use of structural and interactive parent-child teaching approaches in establishing their child’s health practices. In this study, the gender of the participants was homogeneous as all were female. This is an important factor to be considered in future research regarding gender differences in father’s health teaching experience and capacity. Extending the current research to include diverse samples in ethnicity and/or socio-economic class would be a valuable next step. Finally, the sequence of the data collection may have altered or impacted upon the mothers’ health practices, because the interviews were conducted before the observation of the teaching session.

## Conclusion

This study has provided a valuable insight into the approaches Hong Kong Chinese mothers use to teach their preschool children to prevent seasonal influenza. The findings identify the critical role of the community nurse in enhancing effective parent-child interaction for children learning health practices. Nurse-parent-child partnerships are an important consideration when planning and implementing seasonal influenza health promotion.
